# STAT3-activated CD36 facilitates fatty acid uptake in chronic lymphocytic leukemia cells

**DOI:** 10.18632/oncotarget.25066

**Published:** 2018-04-20

**Authors:** Uri Rozovski, David M. Harris, Ping Li, Zhiming Liu, Preetesh Jain, Alessandra Ferrajoli, Jan Burger, Phillip Thompson, Nitin Jain, William Wierda, Michael J. Keating, Zeev Estrov

**Affiliations:** ^1^ Department of Leukemia, The University of Texas MD Anderson Cancer Center, Houston, Texas, USA; ^2^ Institute of Hematology, Davidoff Cancer Center, Rabin Medical Center, Sackler School of Medicine, Tel Aviv University, Tel Aviv, Israel

**Keywords:** CLL, CD36, metabolism, STAT3

## Abstract

Although several studies established that unlike normal B cells chronic lymphocytic leukemia (CLL) cells metabolize fatty acids (FA), how CLL cells internalize FA is poorly understood. Because in various cell types CD36 facilitates FA uptake, we wondered whether a similar mechanism is operative CLL. We found that CD36 levels are higher in CLL cells than in normal B cells, and that small interfering RNA, CD36 neutralizing antibodies or sulfosuccinimidyl oleate (SSO) that inhibits CD36 significantly reduced the oxygen consumption of CLL cells incubated with FA. Because CD36 is oeverexpressed and STAT3 is constitutively activated in CLL cells, we wondered whether STAT3 induces CD36 expression. Sequence analysis identified putative STAT3 binding sites in the CD36 gene promoter. Chromatin immunoprecipitation and an electrophoretic mobility shift assay revealed that STAT3 binds to the CD36 gene promoter. A luciferase assay and STAT3-small hairpin RNA, that significantly decreased the levels of CD36 in CLL cells, established that STAT3 activates the transcription of the CD36 gene. Furthermore, SSO induced a dose-dependent apoptosis of CLL cells. Taken together, our data suggest that STAT3 activates CD36 and that CD36 facilitates FA uptake in CLL cells. Whether CD36 inhibition would provide clinical benefits in CLL remains to be determined.

## INTRODUCTION

Chronic lymphocytic leukemia (CLL) is characterized by a gradual increase in the number of circulating mature appearing lymphocytes. For more than four decades CLL was viewed as a disease characterized by an accumulation of long-lived mature looking neoplastic B-lymphocytes that do not proliferate and do not die. Studies conducted in recent years clearly demonstrated that CLL cells do proliferate [1–3] and undergo spontaneous apoptosis [4]. The proliferation rate of CLL cells varies, however based on heavy water labeling studies it is estimated that approximately 1% of the CLL clone expands daily [5], an expansion rate that is remarkably similar to that of normal adipocytes [6].

We and others [7, 8] have recently shown that, unlike normal B cells, CLL cells adopt metabolic pathways that are operative in adipocytes and myocytes. Specifically, we found that CLL cells store lipids in intracytoplasmic vacuoles and, by aberrantly expressing lipoprotein lipase (LPL), utilize fatty acids (FA) for the production of chemical energy [8, 9]. We have also shown that LPL expression and FA metabolism in CLL is driven by constitutive activation of the signal transducer and activator of transcription (STAT)-3. However, what is the source of FA and what mechanisms are recruited to enable FA entry into CLL cells is not completely understood.

CD36, also known as fatty acid translocase protein, is a multi-ligand glycoprotein that is expressed on the extracellular membrane and facilitates FA uptake in various cells such as myocytes and adipocytes [10] in which lipid uptake is markedly impaired when CD36 function is compromised [11–13]. Because CLL cells’ metabolic pathways resembles those of adipocytes [14] and the CD36 gene harbors putative STAT3-binding sites, we hypothesized that STAT3 induces CD36 cell surface expression and that CD36 facilitates FA uptake in CLL cells.

## RESULTS

### CLL cells express high CD36 protein levels of

**Figure 1:**
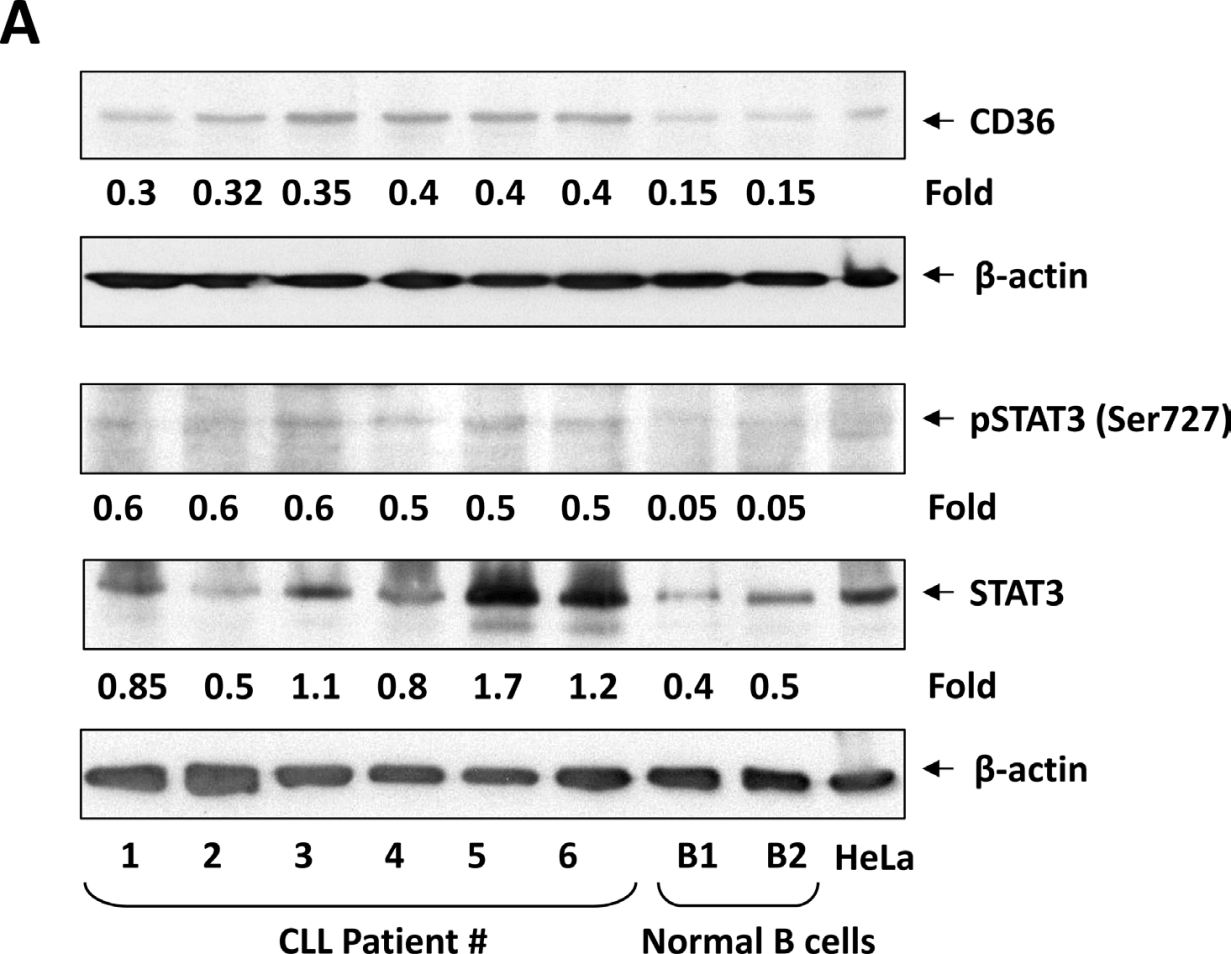

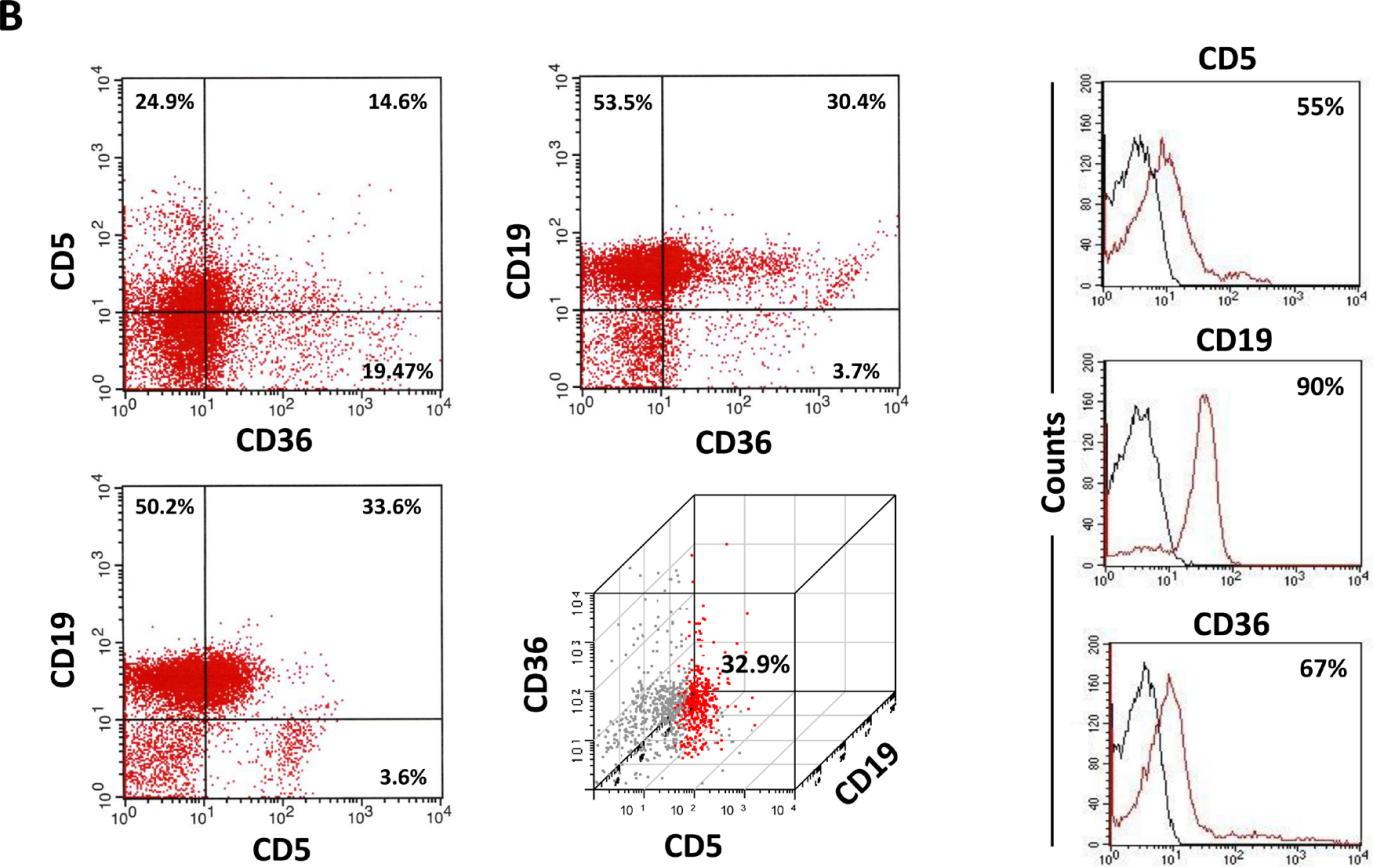

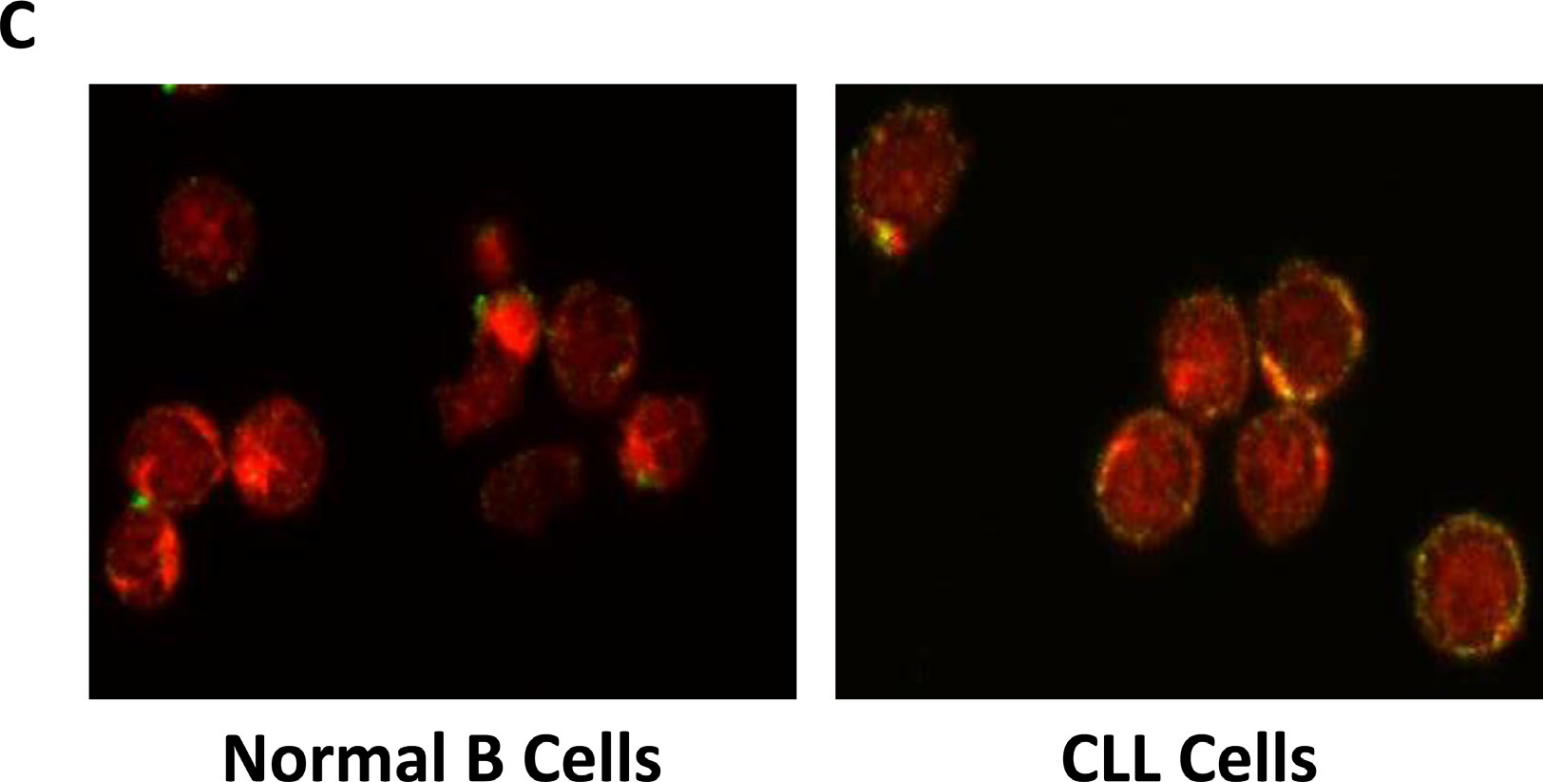
CLL cells express high levels of CD36 (**A**) Western blot analysis of CLL cells from 6 different patients and CD19+ normal B cells from 2 normal controls. In all patients samples CD36 was detected at higher levels than in normal B cells. Phosphoserine STAT3 was detected in all patient samples but not in CD19+ normal B cells and levels of unphosphorylated STAT3 were higher in CLL patient samples than in CD19+ normal B cells. Actin was used as loading control and densitometry units were normalized to actin units in the corresponding lanes. (**B**) CLL cells co-express CD19, CD5 and CD36. Left panel: A representative figure of flow cytometry analysis performed on CLL cells from 6 different patients is depicted. Right panel: Isotype controls. The black line is Isotype control and the red line is the corresponding antibody. Percentages represent the differences between the isotype and the specific antibody. The precentages are the (**C**) Confocal microscopic images (×400) of freshly isolated CLL cells (right) and normal B cells (left) stained with anti-CD36 antibodies (green) and Evans blue (red) showing CD36 on the cell surface of CLL cells but not on normal B cells.

To assess the levels of CD36 protein in CLL cells we performed Western blot analysis of PB-derived CLL cells from 6 randomly chosen CLL patients and, as control, CD19+ B cells from the PB of two healthy individuals. As shown in Figure 1A, protein levels of CD36 were readily detected in all CLL-patient samples and their levels were significantly higher than those of CD19+ B cells obtained from healthy individuals. As in previous studies [15], phosphoserine STAT3 was detected in CLL cells but its levels were undetectable in normal CD19+ B cells. To confirm that CLL cells express CD36 cell membrane protein we performed flow cytometry and detected CD36 protein on the surface of PB CLL cells from 6 randomly selected CLL patients (Figure 1B). However, the percentages of cells co-expressing CD19, CD5, and CD36 antigens varied among the different patients and were 14, 25, 32.9, 37, 41, and 51, respectively. To confirm these findings we used confocal microscopy and found that, like in other cell types, CD36 is detected on the surface but not in the cytoplasm or nucleus of CLL cells (Figure 1C).

### STAT3 binds to the CD36 gene promoter and activates the CD36 gene

**Figure 2:**
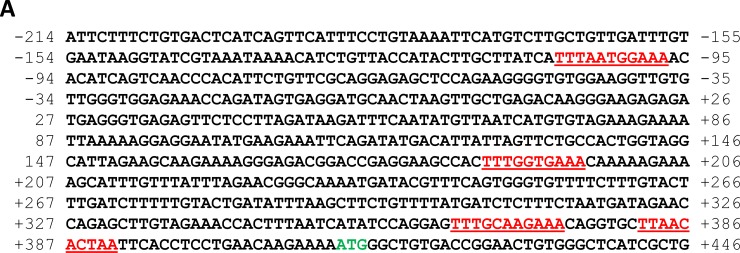

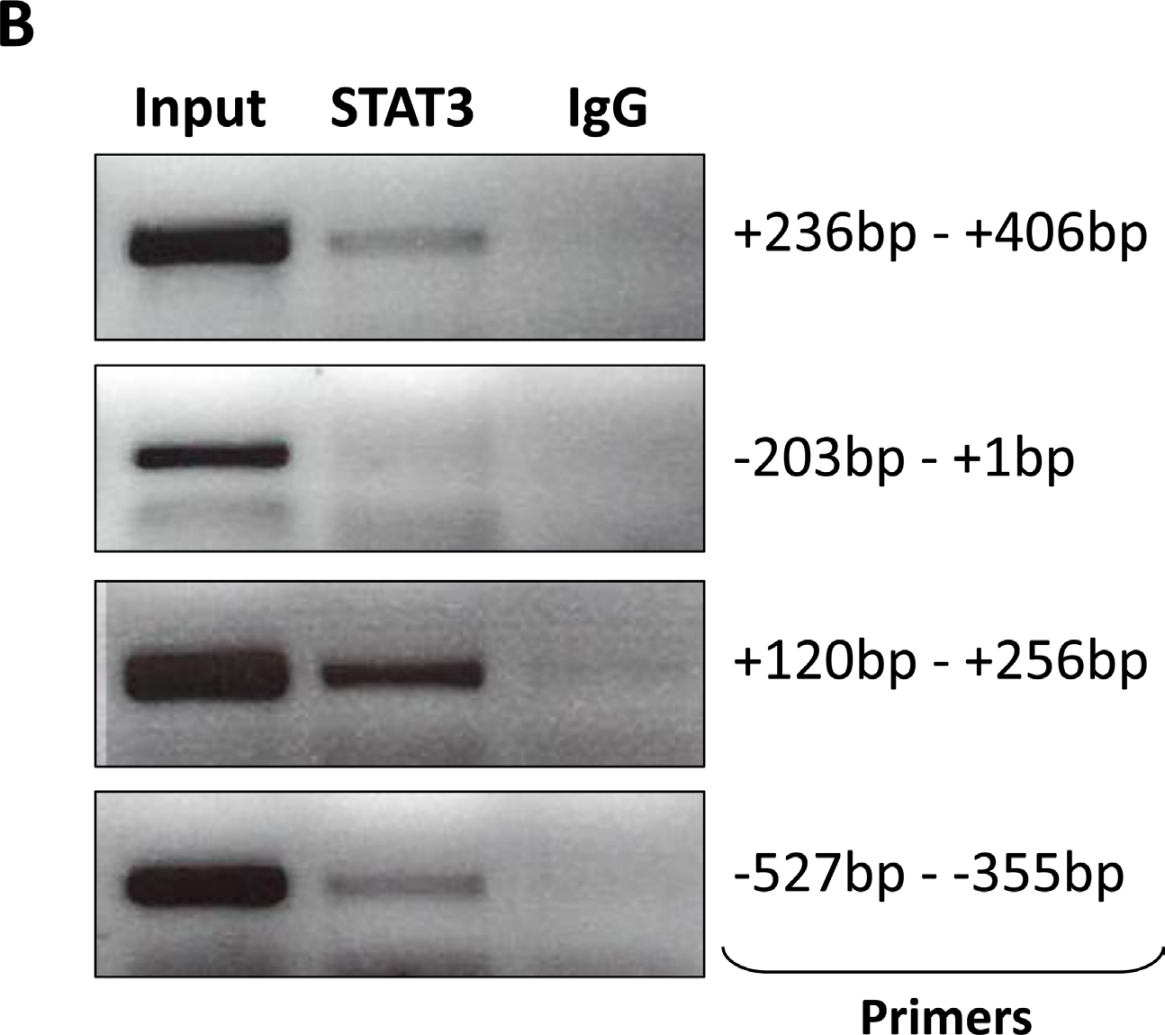

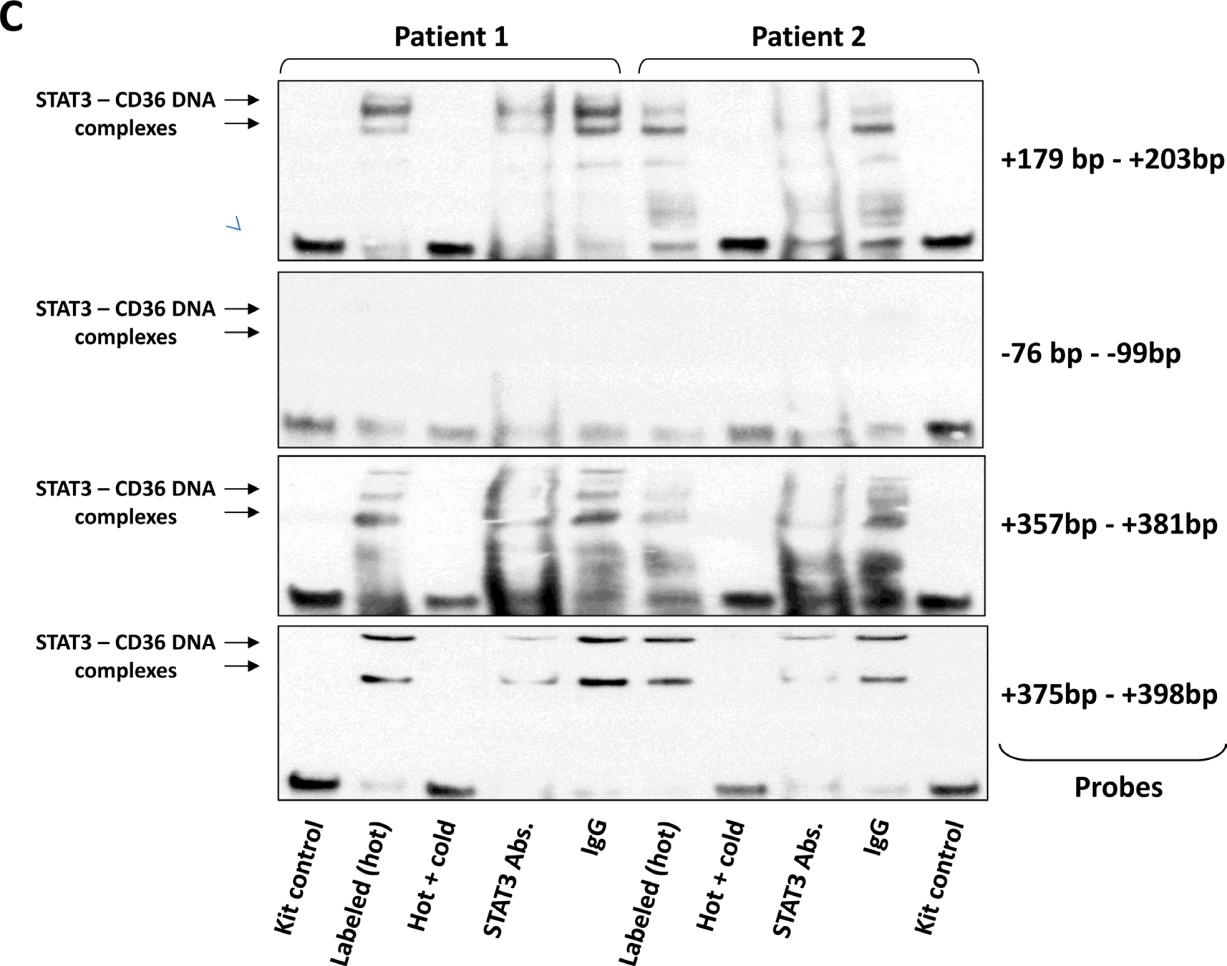

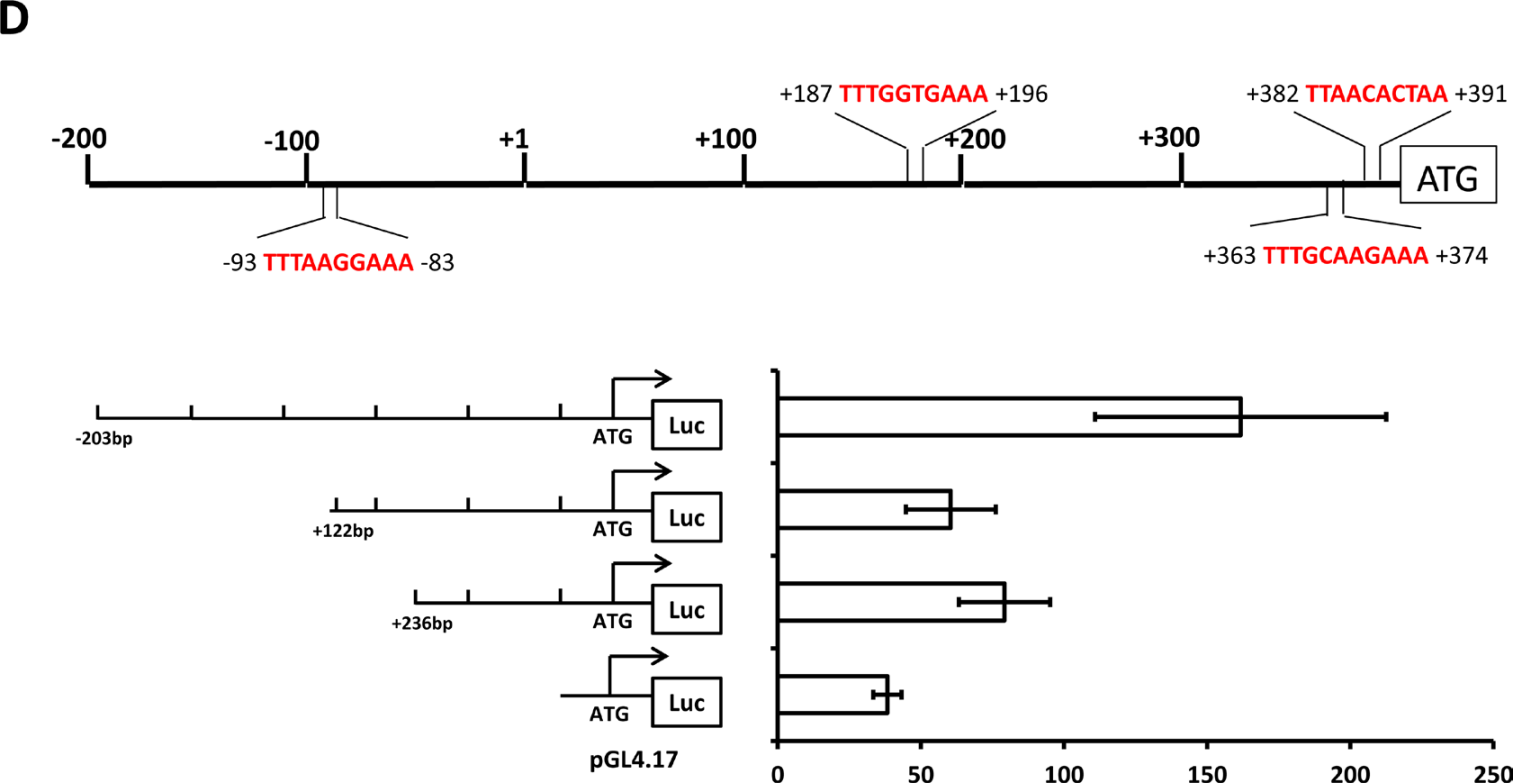

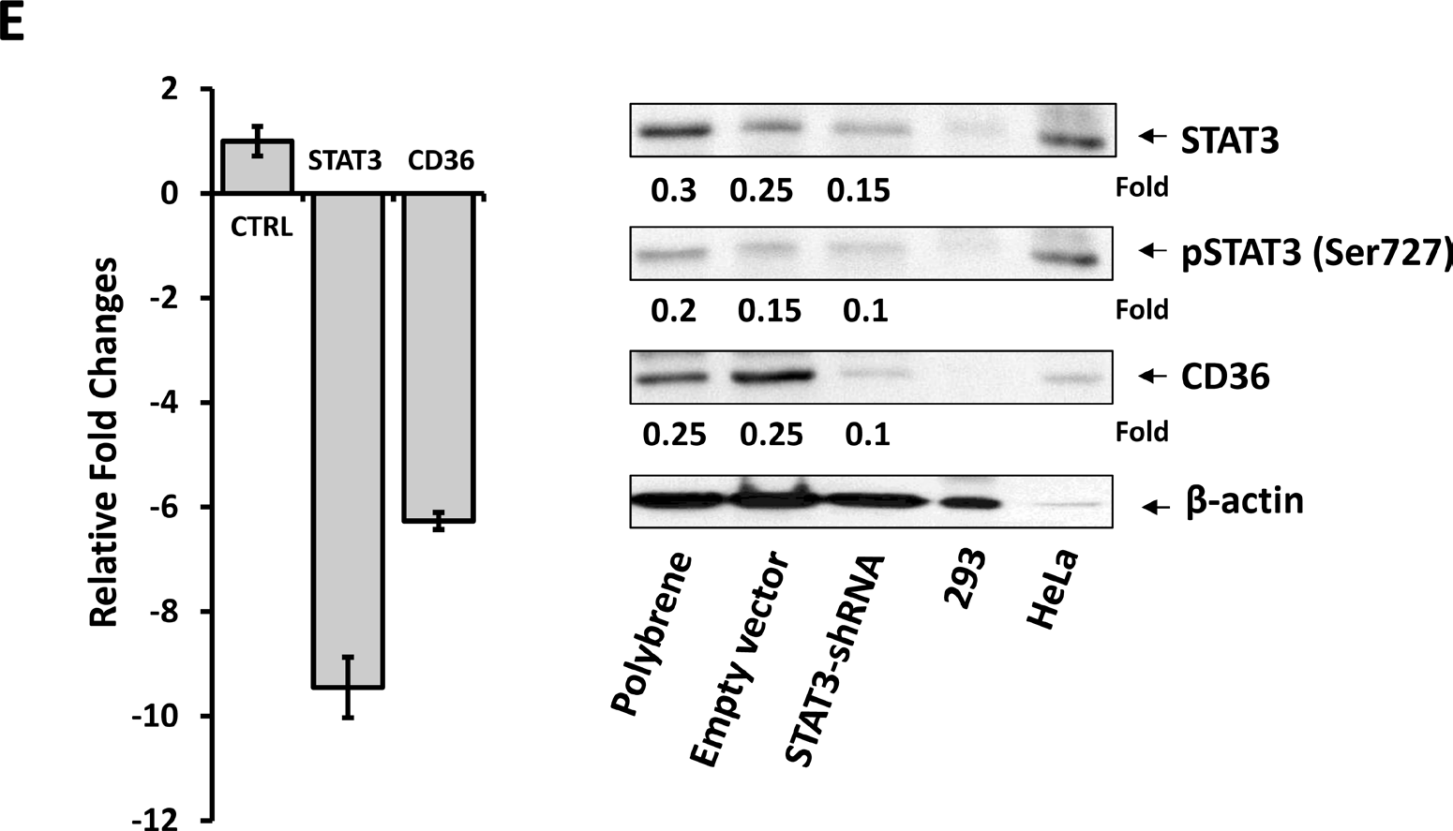
STAT3 binds to and activates the CD36 gene promoter (**A**) Sequence analysis of the CD36 gene promoter upstream of the start codon (green) revealed 4 putative STAT3 binding sites (red). Upstream putative STAT3 biding sites are not shown because in most experiments we concentrated our efforts on studying binding sites that are in proximity to the *CD36* start codon. (**B**) Using the ChIP method, CLL-cell chromatin fragments pulled down by anti-STAT3 antibodies were analyzed by PCR using primers directed at the 4 putative STAT3 binding sites upstream the CD36 gene start codon. As shown, anti-STAT3 antibodies co-immunoprecipitated the DNA detected by primers +236 bp – +406 bp, +120 bp – +256 bp (amplifying regions of the STAT3 putative binding sites +187 bp – +196 bp, +363 bp – 374 bp, and +382 bp – +391 bp), and −527 bp – −355 bp but not by primers −203 bp – +1 bp (amplifying the region of the putative binding site −93 bp – 83 bp). (**C**) Using EMSA, biotin-labeled CD36-DNA probes were incubated with CLL cells’ protein extract from 2 patients. The EMSA demonstrated that CLL cell nuclear protein extracts bound to the CD36 gene promoter at regions that include the putative STAT3 binding sites +187 bp – +196 bp, +363 bp – +374 bp, and +382 bp – +391 bp, but not the region that includes the putative binding site −93 bp – 83 bp, and that the addition of excess unlabeled probe or anti-STAT3 antibodies attenuated the binding. (**D**) The luciferase activity of IL-6-stimulated MM-1 cells was assessed 24 hours after transfection with the 3 depicted DNA fragments containing putative STAT3 binding sites in close proximity to the start codon is shown. The luciferase activity of each of the human CD36 promoter constructs was determined by calculating the constructs’ luciferase activity relative to the activity of the Renilla luciferase produced by the pRL-SV40 control vector. The luciferase activity of unstimulated MM1 cells (not shown) was similar to that of the pRL-SV40 control vector. The highest luciferase activity, compared to the pGL4.17 (control), in IL-6-stimulated MM1 cells transfected with the promoter fragment that included the 3 active binding sites (−203 bp; *P* = 0.0002). A lower albeit increased activity was observed in cells transfected with the promoter fragment that included 2 active binding sites (+236 bp; *P* = 0.009). There was no significant difference in the luciferase activity of fragments +122 bp and +236 bp (*P* = 0.12). (**E**) Infection of CLL cells with STAT3-shRNA, downregulated mRNA levels both of STAT3 and CD36 by 9 and 6 fold, respectively (left panel), and significantly reduced protein levels of STAT3, phsophoserine STAT3 and CD36 (right panel). The figure depicts representative results of 3 different experiments.

Because STAT3 is constitutively activated in CLL cells [15], we wondered whether overexpression of CD36 is driven by STAT3. Using the TFSEARCH database [16] we identified 4 GAS-like elements, known as putative STAT3-binding sites [17], within 600 bp upstream of the CD36 gene start codon (Figure 2A). To determine whether STAT3 binds to any of these GAS-like elements we performed ChIP. Our ChIP analysis revealed that 3 DNA fragments, whose primers amplified the regions of the putative STAT3 binding sites +187 bp – +196 bp, +363 bp – +374 bp, and +382 bp – +391 bp, but not the region of the putative STAT3 binding site −93 bp – −83 bp, co-immunoprecipitated with anti-STAT3 antibodies (Figure 2B). To confirm that these DNA fragments bind STAT3 we performed EMSA using biotinylated DNA probes corresponding to the binding sites +187 bp – +196 bp, +363 bp – +374 bp, and +382 bp – +391 bp, and −93 bp – −83 bp. Confirming the ChIP data, we found that CLL cell nuclear protein extracts from 2 different patients formed complexes with STAT3 binding sites +187 bp – +196 bp, +363 bp – +374 bp, and +382 bp – +391 bp, but not with the putative binding site −93 bp – −83 bp and that the binding was significantly attenuated by the addition of excess unlabeled probe or by anti-STAT3 antibodies (Figure 2C). To further delineate these findings we performed a luciferase assay of MM1 cells in which we concentrated on putative STAT3 binding sites that were in close proximity to the CD36 gene start codon. We found the highest luciferase activity in IL-6 stimulated MM1 cells transfected with the promoter fragment that included the 3 active binding sites (Figure 2D). Furthermore, we found that STAT3-shRNA downregulated mRNA levels both of STAT3 and CD36 by 9 and 6-fold, respectively, and as a result, significantly reduced STAT3 and CD36 protein levels, confirming that STAT3 induces the expression of CD36 in CLL cells (Figure 2E).

### CD36 facilitates FA intake and metabolism in CLL cells

**Figure 3:**
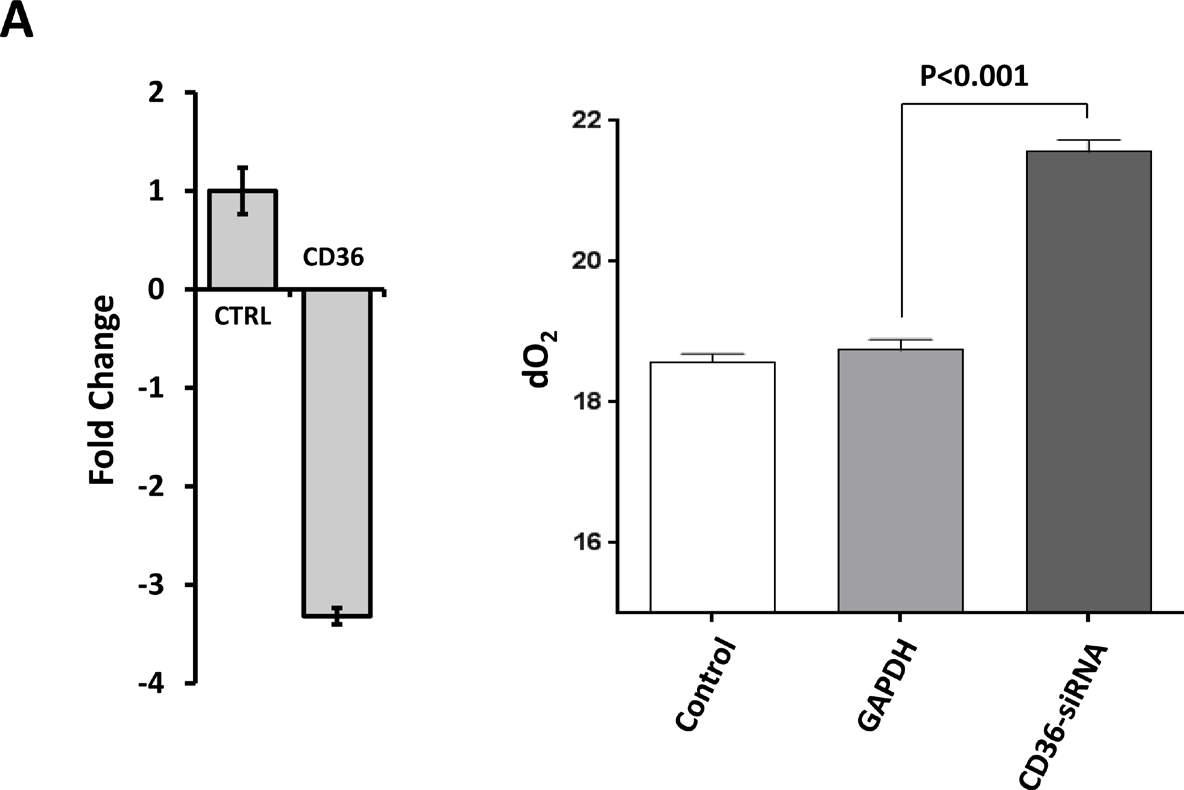

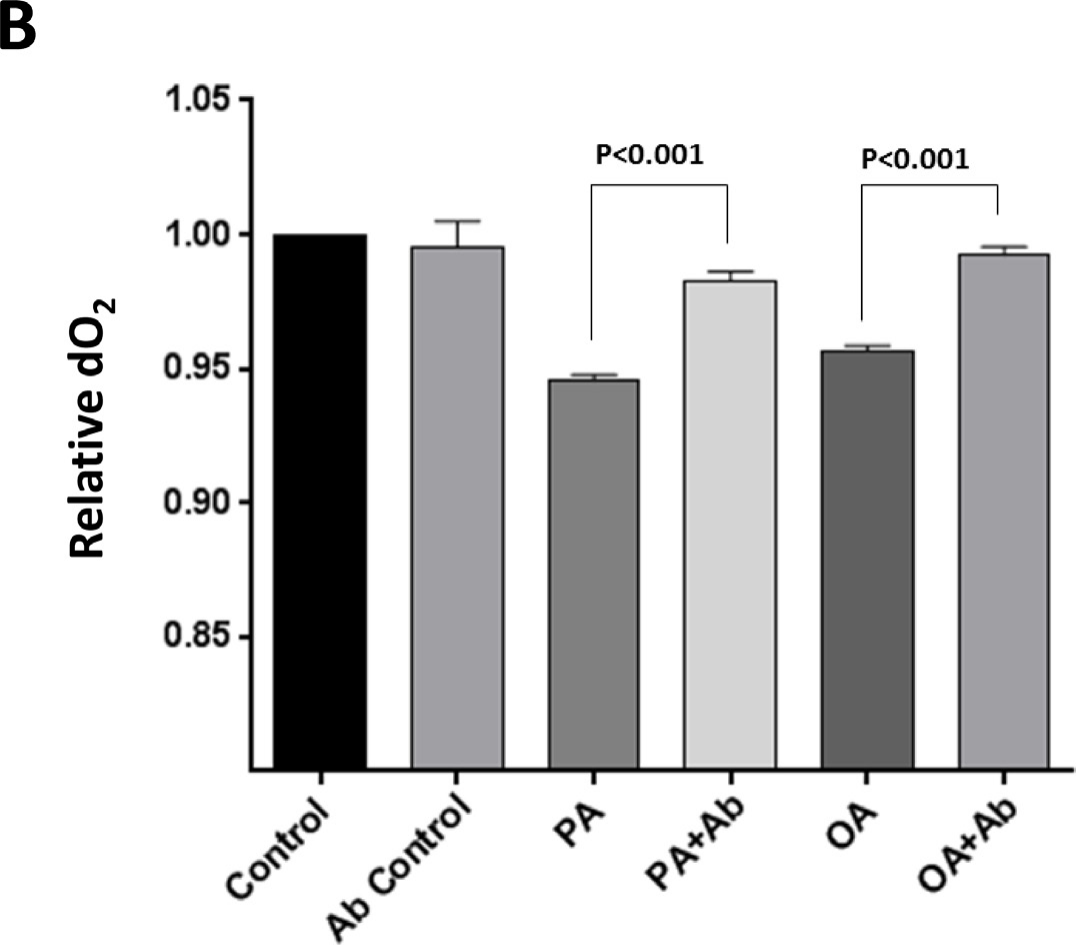

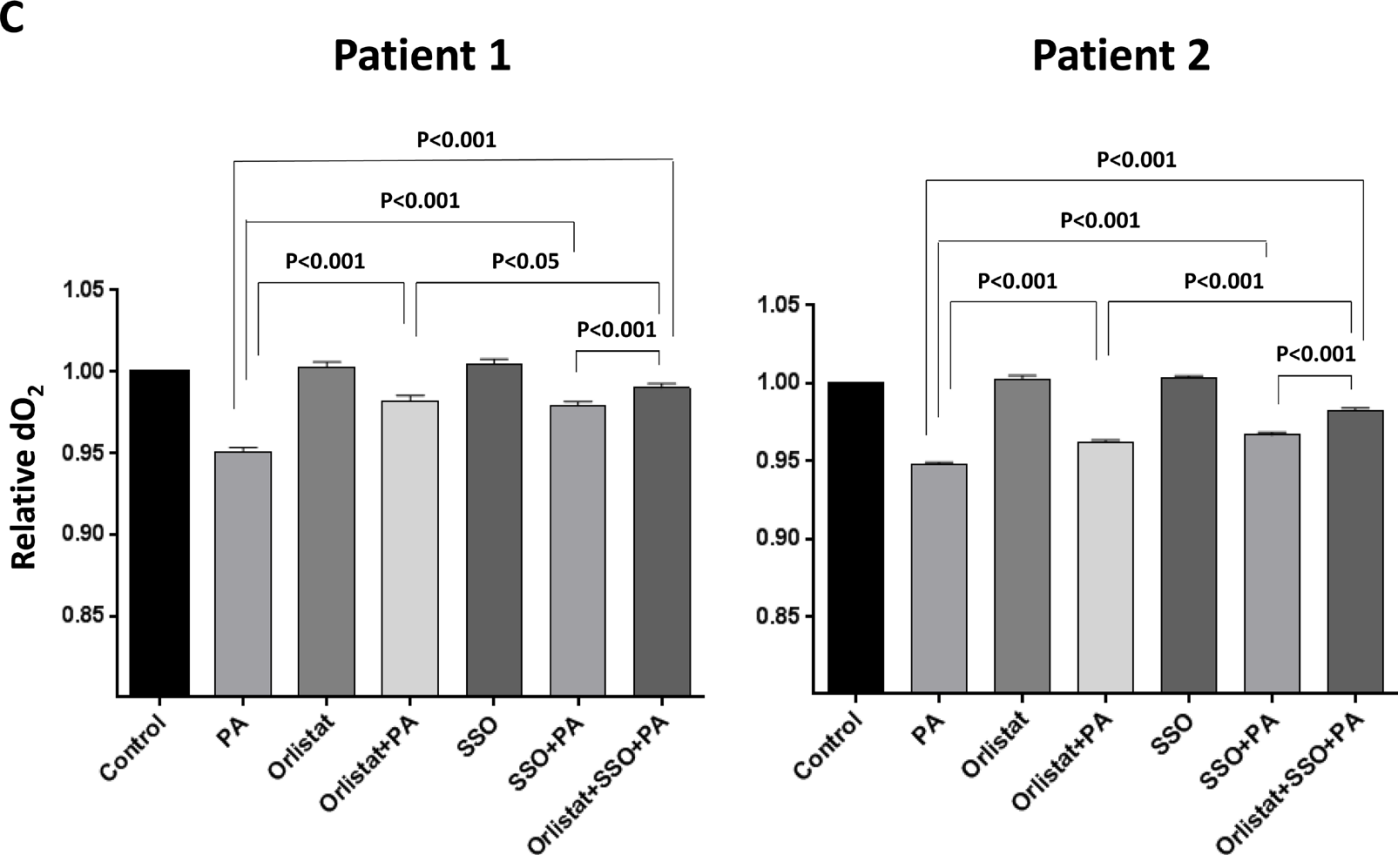

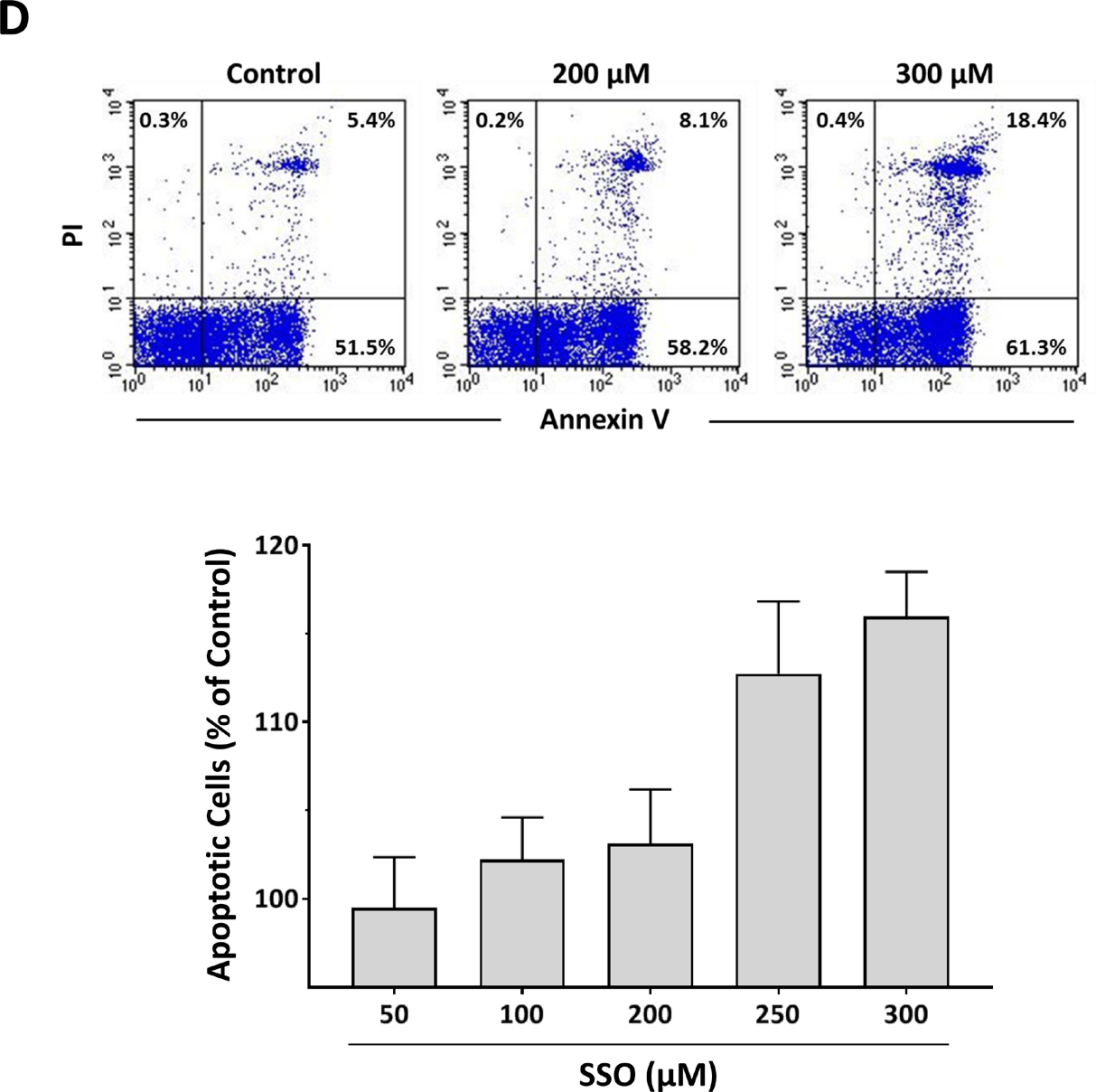
CLL cell FA intake and metabolism is CD36-dependent (**A**) CLL cells were transfected with CD36-siRNA or GAPDH and incubated with 80 mM oleic acid in a serum-free, glucose-free medium in tightly sealed flask for 48 hours. Transfection (at a transfection efficiency of 30% as assessed by flow cytometry; left panel) with CD36-siRNA, but not GAPDH, significantly reduces CD36 mRNA levels compared to transfection with GAPD (CTRL), and the dO_2_ levels were significantly reduced in the medium of non-transfected or GAPDH-transfected CLL cells but not in the medium of CD36-siRNA-transfected cells (right panel). Representative results from 3 different experiments are depicted. (**B**) CD36 neutralizing antibodies inhibited FA metabolism in CLL cells. CLL cells from two patients were incubated in the presence or absence of 80 mM palmitic acid (PA) or oleic acid (OA) with or without CD36 neutralizing antibodies (Ab) in sealed tissue culture flasks. The relative dO_2_ in the culture media was assessed prior to and after 48 hours of incubation. All control cultures contained ethanol at the same concentration as in palmitic acid and oleic acid. To compare dO_2_ we assessed the difference in O_2_ concentration before and after the incubation. The relative difference in dO_2_ before after adding FA is depicted after the dO_2_ in the controls were set to 1. As shown, the CD36-neutralizing antibodies did not affect the cells’ oxygen consumption whereas in the presence of PA or OA the cells consumed O_2_, and this effect was reversed when neutralizing antibodies were added to culture. (**C**) CLL cells from 2 patients were incubated for 48 hours in the presence or absence of 80 mM palmitic acid (PA) with or without the LPL inhibitor orlistat, the CD36 inhibitor SSO or both. The culture media concentration was assessed prior to and after 48 hours of incubation. As shown in the presence of PA dO_2_ was reduced, but less when SSO or orlistat were added to culture and the effect of SSO and orlistat was additive, indicating that these agents reduced FA-dependent metabolism. (**D**) Apoptosis was assessed in CLL cells incubated with or without increasing concentrations of SSO using flow cytometry with annexin V/PI staining. Upper panel: Representative flow cytometry plots of the below depicted three experiments. Lower panel: Results of 3 different experiments, using cells from 3 different patients are depicted as percent of the apoptosis rate in cells incubated without SSO (control).

Because CLL cells utilize FA and CD36 plays a key role in FA uptake in various cell types [18, 19], we sought to determine whether CD36 contributes to FA uptake in CLL cells. We cultured CLL cells in tightly closed flaks in serum- and glucose-free medium and measured the oxygen concentration prior to and after adding FA, assuming that if the cells consume the FA, oxygen levels in the culture medium will drop. As expected, when FA were added to culture the levels of oxygen dissolved in the culture media were markedly reduced whereas the dO_2_ levels of CLL cells transfected with CD36 siRNA and incubated with oleic acid, remained significantly higher than the dO_2_ levels in the medium of non-transfected or GPDH-transfected CLL cells (Figure 3A). Similarly, the dO_2_ levels of CLL cells incubated with oleic acid or palmitic acid in the absence of CD36 neutralizing antibodies significantly dropped whereas dO_2_ levels of CLL cells incubated with oleic acid or palmitic acid in the presence of CD36 neutralizing antibodies remained unchanged (Figure 3B). Likewise, the irreversible CD36 inhibitor SSO and the LPL inhibitor orlistat significantly reduced CLL cell O_2_ consumption and the effect of both inhibitors was significantly additive (Figure 3C). Furthermore, SSO induced apoptosis of CLL cells in a dose-dependent manner (Figure 3D).

## DISCUSSION

Here we show that CLL cells express high levels of CD36 cell surface protein, that overexpression of CD36 is driven by STAT3-mediated activation of the CD36 gene, and that CD36 facilitates uptake of FA by CLL cells.

Although CLL is a heterogeneous disease, in all patients, regardless of clinical characteristics, disease burden, cytogenetic abnormalities or IgHv gene mutation status, STAT3 is constitutively phosphorylated on serine 727 residues [15, 20]. Phosphoserine STAT3 activates a diverse repertoire of coding and non-coding genes which protect CLL cells from apoptosis and provide the cells with proliferation capacity [8, 21–23]. The common constitutive activation of STAT3 in all CLL patients suggests that STAT3 is involved in a core function that affects CLL cell survival.

Several observations linked STAT3 to cellular metabolism in malignant and non-malignant cells [24]. In cancer cells STAT3 emerged as a as a key regulator of metabolism that integrates signals via both mitochondrial and nuclear activities [25]. We have recently shown that by inducing LPL production, STAT3 promotes uptake and metabolism of lipid particles [8]. In various cell types including CLL cells, lipid particles are stored in vacuoles and following additional processing triglycerides undergo hydrolysis into FA generating substrates that enter the Krebs cycle. Here we show that CLL cells utilize a complementary strategy for a direct uptake of circulating FA by STAT3-mediated expression of CD36.

Studies in CD36-null mice identified CD36 as a facilitator of FA uptake [26]. In addition, several groups reported that CD36-mediated FA uptake plays a role in the pathogenesis of various neoplasms. For example, Nath *et al*. showed that in hepatocellular carcinoma CD36-mediated uptake of FA induces epithelial to mesenchymal transition and metastasis [27]. Likewise, CD36-mediated FA uptake was found to promote metastatic development in a subpopulation of human oral carcinoma cells that express high levels of CD36 [28].

To evaluate the role of CD36 in CLL cells we used three different strategies. We downregulated CD36 mRNA levels by transfecting CLL cells with CD36 siRNA and, in other experiments, blocked CD36 activity with CD36-neutralizing antibodies or SSO. Using these three different methods we consistently found that inhibition of CD36 disrupted FA metabolism, confirming that in CLL cells CD36 is required for FA uptake.

Sulfo-N-succinimidyl oleate (SSO) attaches to the CD36 binding pocket and, by doing so, it inhibits CD36-mediated FA uptake [29]. Therefore SSO has been extensively used to study the role of CD36. For example, SSO was found to effectively block CD36-mediated fatty acid uptake into cardiomyocytes [11, 30, 31] and to restore of diabetic heart's function following hypoxia/reoxygenation [32]. However, SSO is chemically instable and therefore cannot be used in its current form for long-term experiments or *in vivo* studies [11]. The use of CD36 neutralizing antibodies is an attractive alternative that has been used in animal models [31, 33]. CD36 neutralizing antibodies were shown to eliminate lymph node metastasis in mice that were inoculated with tumor cells [28], suggesting that it may also be applicable in treatment of CLL-affected lymph nodes.

In conclusion, unlike their normal resting B cell counterparts, CLL cells utilize FA. FA uptake in CLL cells is facilitated by STAT3-enhanced CD36 expression. Whether inhibition of CD36-dependent FA uptake might have therapeutic benefit in CLL remains to be determined.

## MATERIALS AND METHODS

### Patients’ characteristics

Peripheral blood samples were obtained from 19 patients with CLL who were treated at The University of Texas MD Anderson Cancer Center Clinic. The study was approved by our Institutional Review Board and patients’ Informed Consent was obtained prior to sample collection. Clinical characteristics of all patients that participated in this study are depicted in Table 1.

**Table 1:** Patient characteristics (*n* = 25)

Characteristic	Measure/Category	Overall
**Age, years**	Median (range)	58 (40–78)
**WBC** × 10^9^/L	Median (range)	27 (7–55)
**ALC** × 10^9^/L	Median (range)	22 (1–48)
**Rai stage**	(0, 1–2/3–4) (%)	(95/5)
**CD38**	≤30/>30 (%)	84/16 (%)
**Zap-70**	Negative/Positive	78/22 (%)
**β2M (mg/L)**	(</≥ 4 mg/L)	95/5 (%)
**IGHV mutation**	(M/UM)	78/22 (%)
**FISH result, *n***	del17p/11q/T12/13q/Negative	1/3/3/10/2
**Karyotype, *n***	Diploid/Non-diploid/Complex/not done	11/7/0/1
**Survival status,(*n***)	Alive/Dead	19/0

WBC, white blood cell count; ALC, absolute lymphocyte count; β2M, beta-2 microglobulin; M, mutated; UM, unmutated; FISH, fluorescence *in situ* hybridization.

### Cell fractionation

PB cells were fractionated using Ficoll Hypaque 1077 (Sigma, St. Louis, MO). The low-density cellular fraction was used immediately or frozen for additional studies. More than 95% of the peripheral blood lymphocytes obtained from these patients were CD19+/CD5+, as assessed by flow cytometry (Becton, Dickinson and Company, Franklin Lakes, NJ). As control studies we obtained from the Central Blood Bank left-over buffy coats of healthy blood donors. After Ficoll-Hypaque fractionation, the donors’ B cells were isolated using Miltenyi CD19-coated beads according to the manufacturer's instructions (Miltenyi Biotec, Bergisch Gladbach, Germany).

### Western immunoblotting

Western immunoblotting was performed as previously described [34]. Briefly, CLL cell extract was prepared. The protein concentration was determined using a Micro BCA protein assay reagent kit (Thermo Scientific, Pierce, Rockford, IL). Cell lysates were denatured and following electrophoresis transferred to a nitrocellulose membranes. The membranes were incubated with either monoclonal mouse anti-human STAT3 (BD Bioscience, Palo Alto, CA), polyclonal rabbit anti-human phosphoserine (serine 727) STAT3 (Cell Signaling Technology, Beverly, MA), or rabbit anti-human CD36 (Pierce, Thermo Scientific, Waltham, MA) antibodies and Horseradish peroxidase-conjugated secondary antibodies (GE Healthcare, Amersham, Buckinghamshire, UK). Proteins were visualized via an enhanced chemiluminescence detection system (GE Healthcare).

### Flow cytometry analysis

CLL cells were fixed in 2% paraformaldehyde for 10 minutes and permeabilized overnight at −20° C. Before staining cells were washed three times in PBS with 2% FBS. Cells were then stained with CD19 (BD Biosciences, San Jose CA), CD5 (BD Biosciences), and CD36 (BD Biosciences) as well as appropriate isotypic controls. Cells were analyzed on a FacsCaliber flow cytometer (BD Biosciences) and data analysis performed using CellQuest software (BD Biosciences). Graphics were created with CellQuest (BD Biosciences) and WinList (Verity Software House, Topsham, ME) software.

### Confocal microscopy

A previously described method was used [35]. CLL low-density cells were incubated in microtubes in PBS supplemented with 5% bovine serum albumin (Cell Signaling Technology,). After 1 hour of incubation, the cells were washed three times with PBS and then incubated with mouse anti-CD36 antibodies (Novus Scientific, Littleton, CO) for 1 hour, washed in PBS, re-suspended in a 0.1% solution of Evans blue dye (Sigma-Aldrich) for 5 minutes, and washed in PBS to remove unbound dye. The cells were resuspended in PBS and placed into μ-slide VI^0.4^ chamber slides (ibidi LLC, Verona, WI) for microscopic analysis. The slides were viewed using an Olympus FluoView 500 Laser Scanning Confocal Microscope (Olympus America, Center Valley, PA), and images were analyzed using the FluoView software (Olympus America).

### Chromatin immunoprecipitation (ChIP) assay

A chromatin immunoprecipitation (ChIP) assay was performed using a SimpleChIP Enzymatic Chromatin IP Kit (Cell Signaling Technology, Boston, MA) according to the manufacturer's instructions. Briefly, cells were cross-linked with 1% formaldehyde for 10 minutes at room temperature and then harvested and incubated on ice for 10 minutes in lysis buffer. Nuclei were pelleted and digested with micrococcal nuclease. Following sonication and centrifugation, sheared chromatin was incubated with anti-STAT3 or rabbit serum (negative control) overnight at 4° C. Then, protein G beads were added, and the chromatin was incubated for 2 hours in rotation. Antibody-bound protein-DNA complexes were eluted and subjected to PCR analysis. The primers to amplify the human CD36 promoter were F’: −201 and R: +1, which generate a 200-bp product that covers the GAS binding site-93 to −83 bp upstream of *CD36*; F’: +120 and R’: +256, which generate a 136-bp product that covers the GAS binding site +187 to +196 bp upstream of *CD36*; F’: +236 and R’: +406, which generate a 170-bp product that covers the GAS binding site +363 to +374 bp upstream of *CD36*; and F’: +355 and R’: +427, which generate a 72-bp product that covers the GAS binding site +382 to +391.

### Electrophoretic mobility shift assay (EMSA)

Non-denatured cellular nuclear extracts were prepared using a NE-PER extraction kit (Thermo Scientific Pierce, Rockford, IL). Nuclear protein extracts were incubated with biotin-labeled CD36 promoters’ DNA probes in binding buffer for 30 minutes on ice. All probes which target GAS binding sites were synthesized by Sigma-Genosys (The Woodlands, TX). Following incubation, the samples were separated on a 5% polyacrylamide gel, transferred onto a nylon membrane, and fixed on the membrane via ultraviolet cross-linking. The biotin-labeled probe was detected with strepavidin-horseradish peroxidase (Gel-Shift Kit; Panomics, Fremont, CA). The control consisted of 7-fold excess unlabeled cold probe. To test the effect of STAT3, anti-STAT3 antibodies (BD Bioscience) mouse IgG1 (BD Bioscience) were added with the nuclear extracts [15, 36].

### Transfection of MM-1 cells with CD36 gene promoter fragments and luciferase assay

Four different CD36 promoter fragments, corresponding to the above described putative STAT3-binding sites, were transfected into MM-1 cells by using electroporation. MM-1 cells were used because in these cells STAT3 phosphorylation is induced by extracellular signals such as IL-6. Each construct harbored a luciferase reporter gene and a CD36 promoter fragment that included at least 1 γ-interferon activation sequence (GAS)-like elements. The luciferase activity of unstimulated or IL-6-stimulated MM-1 cells was assessed 24 hours after transfection using a Dual-Luciferase Reporter Assay System (Promega) and a Sirius luminometer V3.1 (Berthold Detection Systems, Pforzheim, Germany). The luciferase activity of each of the human CD36 promoter constructs was determined by calculating the constructs’ luciferase activity relative to the activity of the Renilla luciferase produced by the pRL-SV40 control vector.

### Generation of GFP-conjugated lentiviral STAT3 short hairpin RNA (shRNA) and infection of CLL cells

293T cells were co-transfected with GFP-conjugated lentiviral STAT3 shRNA or a GFP-conjugated empty lentiviral vector and with packaging vectors (pCMV delta R8.2 and pMDG generously provided by Dr. Giorgio Inghirami, (department of pathology, University of Torino, Italia) using the Superfect transfection reagent (QIAGEN Inc.) as previously described [15]. The 293T cell culture medium was replaced after 16 hours and collected after 48 hours. Then, the culture medium was filtered through a 45-μm syringe filter to remove floating cells, the lentivirus was concentrated by filtration through an Amicon Ultra centrifugal filter device (EMD), and the concentrated supernatant was used to transfect CLL cells. CLL cells (5 × 10^6^/ml) were incubated in six-well plates (Becton, Dickinson and Company, Franklin Lakes, NJ) in 2 mL of Dulbecco's modified Eagle medium (DMEM) supplemented with 10% fetal bovine serum (FBS) and were transfected with 100 μl of viral supernatant. Polybrene (10 ng/mL) was added to the viral supernatant at a ratio of 1:1000 (v/v). Transfection efficiency was measured after 48 hours and ranged between 40% and 50% (calculated on the basis of the ratio of propidium iodide–negative and GFP-positive cells). These experiments were conducted using an upgraded FACSCalibur flow cytometer (Becton, Dickinson).

### Transfection of CLL cells with CD36 small interfering RNA (siRNA)

Hundred μM human CD36-siRNA, 10 μl FAM-labeled siRNA, targeting the human glyceraldehyde 3-phosphate dehydrogenase, or a scrambled control (Applied Biosystems, Foster City, CA) were added to 10 μl siPORT NeoFX transfection reagent diluted in 50 μl Opti-MEMI reduced serum medium (Thermo Fisher Scientific), and incubated at room temperature for 10 minutes. The transfection agents were incubated at room temperature with 1 × 10^7^ CLL cells suspended in 0.2 ml Opti-MEM I medium. After 1 hour of incubation, electroporation (Bio-Rad Laboratories) was performed and the cells were incubated in RPMI supplemented with 10% FBS for 24 hours. Transfection efficiency of the FAM-conjugated siRNA was assessed on a FACSCalibur flow cytometer (Becton Dickinson Biosciences).

### Measurement of cellular O_2_ consumption

Because FA metabolism increases O_2_ consumption, palmitic acid and oleic acid utilization was assessed by measuring the level of dissolved O_2_ (dO_2_) using the SevenGo pro Dissolved Oxygen Meter (Mettler Toledo, Worthington Columbus, OH).

Preliminary experiments designed to test FA consumption that used palmitic acid or oleic acid dissolved in ethanol, determined that the O_2_ consumption with 80 mM oleic acid or 80 mM palmitic acid (both from Sigma-Aldrich) each is maximal therefore we used these concentrations in the following experiments. In each experiment we used CLL cells at a concentration of 2 *×* 10^6^ cells/ml. The cells were incubated with a minimum essential medium (MEM) with Hank's salts and L-glutamine (Life Technologies, Carlsbad, CA) or with phosphate buffered saline medium (Invitrogen) for 48 to 72 hours in tightly sealed T25 tissue culture flasks (Corning, Tewksbury, MA ) at 37° C in the presence or absence of palmitic acid or oleic acid. The O_2_ meter probe was placed in the flask and the reading allowed stabilizing. Then, the dissolved O_2_ (dO_2_) level was recorded. The probe was cleaned before it was re-used. Measurements of dO_2_ were repeated at least three times for every data point. The dO_2_ of CLL cells transfected with CD36 siRNA, or incubated either with 2500 mg/ml CD36-neutralizing antibodies (Abcam), with 100 μM of the LPL inhibitor orlistat, or 100 μM of the CD36 inhibitor sulfosuccinimidyl oleate sodium (SSO) was assessed.

We used the Student *t*-test when one experimental condition was compared to non-treated (controls) and one-way ANOVA when 2 experimental conditions were compared to controls. Statistical analyses were performed using GraphPad version 5 (San Diego, California, USA).

### Annexin V/propidium iodide apoptosis assay

The rate of cellular apoptosis was analyzed using double staining with a Cy5-conjugated annexin V kit and propidium iodide (PI; BD Biosciences) according to the manufacturer's instructions. Briefly, 1 × 10^6^ CLL cells were incubated for 24 hours in glucose-free MEM (Life Technologies) supplemented with 10% FCS. After incubation the cells were washed once with phosphate-buffered saline and resuspended in 200 μL binding buffer with 0.5 μg/ml annexin V-Cy5 and 2 μg/ml propidium iodide (PI). After incubation for 10 minutes in the dark at room temperature, the samples were analyzed on a FACSCalibur flow cytometer (Becton Dickinson Biosciences). Cell viability was calculated as the percentage of annexin V-positive cells.
